# Investigating Social Media Use by Young People to Self-Manage Type 1 Diabetes Mellitus: Large-Scale Analysis of Social Media Discussions Using Topic Modeling

**DOI:** 10.2196/78632

**Published:** 2025-10-20

**Authors:** Yanan Ma, Lamiece Hassan, Sabine N van der Veer, Goran Nenadic

**Affiliations:** 1 School of Computer Science University of Manchester Manchester United Kingdom; 2 Division of Informatics, Imaging and Data Sciences University of Manchester Manchester United Kingdom

**Keywords:** self-management, social media, topic modeling, type 1 diabetes, type 1 diabetes mellitus, T1DM, young people, large language model, LLM

## Abstract

**Background:**

Social media has shown promise in supporting young people with type 1 diabetes mellitus (T1DM) by providing information and emotional support. Although previous qualitative studies have investigated young people’s self-reported use of social media for self-management, their patterns of actual use remain underexplored. Furthermore, different platforms may serve different functions or attract different types of engagement, making it important to examine how the patterns of actual use vary across them.

**Objective:**

This study aimed to identify and describe the topics that young people with T1DM discuss online and to identify differences in content across platforms.

**Methods:**

We collected data from Twitter and two forums (Reddit and Diabetes.co.uk) spanning from January 2020 to January 2024, identifying young people with T1DM using rule-based criteria and profile age information. An efficient analysis pipeline, integrating topic modeling with large language model (LLM) summarizing and human verification, was applied to identify the discussion topics.

**Results:**

We analyzed 1765 tweets and 1259 forum posts by young people with T1DM and identified 24 topics. Among these topics, 7 (29.2%) were common across all platforms (Twitter and forums topic sizes, respectively): blood glucose management (n=70, 9%, and n=88, 16.6%), community and friendship (n=33, 4.3%, and n=51, 9.6%), COVID-19 (n=76, 9.8%, and n=13, 2.4%), diabetes and diet (n=81, 10.5%, and n=38, 7.2%), diabetes devices (n=97, 12.5%, and n=128, 24.1%), emotional and psychological expression (n=100, 12.9%, and n=124, 23.4%), and financial challenges (n=141,18.2%, and n=23, 4.3%). In addition, 7 (29.2%) topics were unique to Twitter: advocacy and awareness (n=35, 4.5%), diaversaries reflection (n=24, 3.1%), daily life and adaptation (n=27, 3.5%), misunderstanding of T1DM (n=25, 3.2%), educating and raising awareness (n=19, 2.5%), insulin prescription frustration (n=21, 2.7%), and experience with health care providers (n=25, 3.2%). Furthermore, 3 (12.5%) topics were unique to forums: diabetes complications (n=21, 4%), newly diagnosed and “honeymoon” experiences (n=21, 4%), and traveling with T1DM (n=24, 4.5%).

**Conclusions:**

Although our results confirmed social media’s role in providing information and emotional and peer support, we also found that topics such as COVID-19, advocacy, and diabetes celebration are more frequently discussed in social media than in prior qualitative studies based on self-reporting. Platform-specific patterns are evident, with Twitter discussions being more immediate and experience driven, focusing on daily reflection, advocacy, awareness campaigns, and financial support, while forum-based platforms emphasize more in-depth discussions, where users seek and provide comprehensive advice, troubleshooting, and sustained peer support. Practitioners could consider the differences when designing digital interventions to ensure the delivery method aligns with the communication style and support needs of the target audience.

## Introduction

Type 1 diabetes mellitus (T1DM) is the predominant form of diabetes diagnosed in children and young adults [1], with a worldwide prevalence of 5.9 per 10,000 people per year [2]. Children with T1DM face a reduced life expectancy of approximately 12 years [3,4].

T1DM management is a complex and lifelong process and needs ongoing engagement and support from health care professionals, family, and peers [5,6]. The management activities include tasks such as home blood glucose monitoring, adhering to complicated medical regimens, and psychosocial acceptance of living with T1DM [7,8]. Self-management has been described as a vital component in diabetes prevention and management, thus reducing the incidence of complications and improving diabetes-related quality of life [9-11]. Yet, many young people struggle to self-manage effectively and find it overwhelming [12], while also often suffering from depression, anxiety, stigma, discrimination, and inadequate support [13-16]. Moreover, traditional health care services do not always fully meet their needs because of long waiting hours, scheduling problems, a lack of explanation of treatment, and a fear of being judged [17-20].

Social media platforms have become integral to young people’s daily lives and are increasingly used as a supplement to traditional health care for T1DM self-management. For example, social media serves as an important source of information for acquiring diabetes-related knowledge and skills, empowering users with a better understanding of their condition and its long-term complications [21,22]. Additionally, social media platforms can be a source of emotional support: people who share a condition, such as T1DM, may seek and share peer support, look for validation and sympathy, and thus achieve a sense of normality and belonging among online peers [17,23,24]. The anonymity provided by social media (eg, through pseudonyms) further encourages users to discuss sensitive topics with less fear of embarrassment or judgment, which is more difficult to facilitate in traditional face-to-face interactions [25-27].

Understanding young people’s self-reported and actual use of social media for T1DM self-management is crucial for informing the integration of social media into personalized health care programs and the development of guidelines that enhance diabetes management engagement. Previous qualitative studies have examined young people’s self-reported use of social media for T1DM self-management via interview-based methods [28,29]. Although such approaches provide valuable insights, they do not provide a full picture: qualitative research can be limited by small sample sizes and may not fully capture the experiences of a diverse group of social media users. Furthermore, interview-based studies rely on self-reported data, which may not truly represent actual behaviors because of factors such as recall errors, embarrassment, and lack of insight into their own motivations and behavioral patterns. For example, a previous study [30] showed discrepancies between actual and self-reported social support via Facebook among individuals with depression. This discrepancy can lead to poorly targeted interventions and inefficient use of resources in T1DM health care programs. For instance, programs may be designed around platforms that young people report to use but rarely actually engage with, or may emphasize formal education when instead informal support from peers is more likely to be the first point of call. Furthermore, previous studies [21,28,31] have noted that young people with T1DM may behave differently across platforms due to variations in the audience, visibility, anonymity, community rules, and moderation. Therefore, a large-scale analysis across platforms of young people’s actual use of social media for T1DM self-management is needed to understand a broader range of challenges, emotional states, and self-management practices of young people with diabetes.

To address this gap, this study investigated young people’s actual patterns of use of social media for T1DM self-management based on the content of their online posts and how these differed between platforms.

## Methods

### Data Extraction

In this study, we extracted T1DM-related data from two types of social media platforms: a microblogging site (Twitter, now rebranded as X; throughout this paper, we refer to X as Twitter, as most data were collected before its rebranding to X) and two forum-based platforms (Reddit and Diabetes.co.uk). Twitter, with its 280-character limit, encourages open and concise updates but often lacks detailed textual discussions. In contrast, diabetes-related channels from Reddit (“subreddits”) and Diabetes.co.uk provide spaces for diabetes-specific communities that allow for longer-form, mainly textual content and more comprehensive discussions and are typically moderated. We merged the Reddit and Diabetes.co.uk data, consistently referring to this combined dataset as the *forum-based dataset*. We collected tweets/posts, tweet/post IDs, user IDs, and timestamps across the platforms. For Diabetes.co.uk, we also extracted age information from user profile pages.

We focused on these three platforms due to their accessibility and suitability for text-based content analysis, while excluding platforms such as TikTok, Snapchat, and Instagram, which are primarily visual-first or oriented toward more personal exchanges and therefore less suitable for addressing our study aim.

#### Twitter

We collected data via the official Twitter application programming interface (API) using the following search query: *“type 1 diabetic” OR “type 1 diabetes” OR #T1D OR #T1DM OR #type1diabetes OR #t1diabetes OR #GBDoc OR #T1DLife OR #t1dlookslikeme*. The search was refined to include only original tweets posted in English and excluded retweets to focus on unique perspectives. Data collection was pragmatically limited between January 2020 and May 2023 due to data availability and Twitter’s subsequent API restrictions.

#### Reddit

We collected data on two subreddits (*r/Type1Diabetes* and *r/diabetes_t1*) via the official Reddit API. Due to the limitations of Reddit, we could not extract data in a specific time frame. Instead, we extracted the most discussed posts from the “Hot” category and the latest posts from the “New” category in February 2023, September 2023, and January 2024.

#### Diabetes.co.uk

We collected data from three subforums (*Type 1 Diabetes*, *Children & Teens*, and *Young People/Adults*) on Diabetes.co.uk. We extracted all the available data in September 2023.

#### Young People Identification

We defined young people as those aged between 10 and 26 years, combining the World Health Organization’s definition of young people [32] with the concept of young adulthood proposed by Bonnie et al [33]. For Twitter and Reddit, we used rule-based methods to detect the users’ self-reported age information from their tweets/posts that matched the regular expression patterns (see Multimedia Appendix 1). We then manually labeled the posts to confirm whether the author was within our defined age group. Users were excluded if age indicators were unclear. For example, if a user mentioned having T1DM for 12 years but did not specify their age at the time of posting or diagnosis, we could not calculate whether they were within our age group and excluded them. Three annotators independently labeled the first 200 posts in duplicate to ensure a consistent standard, with the remainder of posts labeled by one annotator. After identifying the users within our defined age group, we used their user IDs to retrieve their additional historical posts, including those that did not contain self-reported age information.

Diabetes.co.uk provided age information in users’ profiles, which we additionally extracted, where provided, to identify young users. We then used their IDs to retrieve additional posts of the users from the extracted posts.

### Data Preprocessing

In our experiment, we excluded duplicate posts and those only containing URLs. We used a publicly available Python library OCTIS [34] to preprocess the posts before analysis. This included removing special characters (eg, hashtags “#,” mentions “@,” and emojis); removing uninformative stop words (eg, “the,” “and,” “of”); conducting lemmazation, which is a process to reduce a word to its base form (eg, turning “running,” “ran,” or “runs” to “run”, turning “best” or “better” to “good”).

### Ethical Considerations

Ethical approval was received from the University of Manchester Research Ethics Committee (reference number 2023-16304-30242). As the data were publicly available and not collected directly from participants and as no intervention was involved, informed consent was not required. Nonetheless, we implemented additional safeguards to protect privacy and confidentiality in line with previous ethical recommendations [35]. To reduce the risk of reidentification, we removed user IDs completely from our dataset prior to analysis. Any posts reported in this paper were rephrased or partially masked to minimize the potential for user identification from “reverse searching.” In addition, we conducted several patient and public involvement (PPI) sessions to review and refine the topic summaries, and participants received a GB £10 (US $13.41) e-voucher as compensation for their time. No images or screenshots containing identifiable user information are included in this manuscript or supplementary materials.

### Topic Identification and Representation

**Figure 1:**
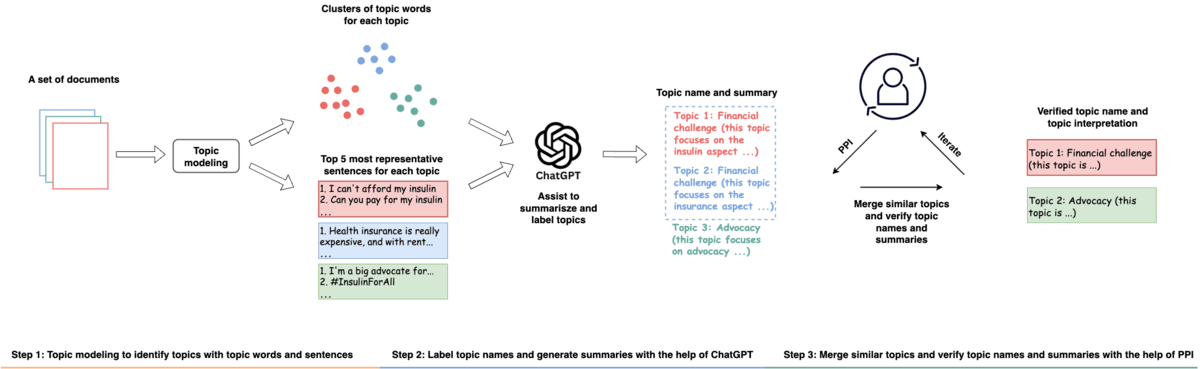
Pipeline for identifying and labeling discussion topics among young people with T1DM. Topic modeling was first used for topic identification. An LLM was then applied to assist with topic representation, and PPI activities were conducted to merge similar topics and refine topic names and interpretations. T1DM: type 1 diabetes mellitus; LLM: large language model; PPI: patient and public involvement.

We developed an analysis pipeline for topic identification and representation, incorporating topic modeling for topic discovery, large language model (LLM)–assisted topic representation, and human-in-the-loop verification, as shown in Figure 1.

More specifically, we first applied topic modeling to identify topics, associated topic words, and the five most representative sentences (selected based on the cosine similarities with the topic vector) for each topic. Second, we labeled and summarized the meaning for each topic based on the topic words and five representative sentences with the help of an LLM (ChatGPT: GPT-4o version). Third, one researcher (author YM), with the help of volunteers with lived experience of T1DM acting in a PPI capacity, manually merged semantically similar topics and refined the topic names and interpretations. For merged topics, we manually selected 10 topic words from the original topic word sets and chose new representative sentences that were highly semantically similar to the original topic.

#### Topic Modeling

Topic modeling is a computational approach to attain valuable insights and detect topics of interest within a large collection of documents. It has been widely used to conduct sentiment analysis, identify public opinions and emerging topics of interest, and detect dynamics of health concerns in the health care domain [36-42]. Existing models include traditional probabilistic models, such as Latent Dirichlet Allocation (LDA) [43]; more advanced embedding-based neural models, such as Top2Vec [44], BERTopic [45], and CAST (which integrates semantic embeddings contextualized on the corpus with clustering and self-similarities to improve topic coherence and interpretability) [46]; and LLM-augmented topic modeling methods [47,48].

In this study, we used CAST, a recent topic modeling method, to identify key topics and representative topic words and sentences. CAST works by jointly encoding documents and words, clustering similar document embeddings, and treating each cluster as a topic. Topic words are those whose embeddings are closest to the cluster center. CAST also uses context from the entire dataset to refine word embeddings and applies a self-similarity score to filter out common or less informative words, reducing the need for manual preprocessing.

We applied CAST to our Twitter and forum-based datasets and found that it outperformed LDA (usually used as a baseline model) in most cases in terms of topic coherence and diversity (see Multimedia Appendix 2 for detailed topic quality evaluation). Based on these results, we used CAST for topic identification and the extraction of representative topic words and sentences.

For implementation, we used the “sentence-transformers/all-mpnet-base-v2” embedding model and set the number of topics to 20. As we did not have prior knowledge of how many topics were in the corpus, we decided to identify the top 20 topics and manually merge the most similar ones. We reduced embedding dimensions to five to balance complexity and interpretability. We set the minimum number of samples to group as a cluster (“min_cluster_size”*)* to five. We adjusted the self-similarity threshold to 0.4 for Twitter and 0.5 for forum data, following CAST guidelines to effectively filter out less informative words. All experiments were conducted on one NVIDIA V100 graphics processing unit (GPU).

#### Topic Representation

LLMs have been widely used in natural language understanding and text summarization [49-51]. In this paper, we used an LLM (ChatGPT: GPT-4o version) to generate topic summaries and assign preliminary topic labels based on the following prompt:

I will provide several sentences related to type 1 diabetes management. Please generate a brief summary and assign an appropriate topic label to the sentences.

One researcher (YM) then reviewed the generated summaries, merged similar topics, and refined the topic names. Topic names and descriptions were then reviewed and refined further by the entire team. For topics where we were less confident about the interpretation, we conducted PPI sessions with four people with T1DM, focusing on sense checking to ensure our understanding of the posts aligned with the experiences of young people with T1DM. Each participant reviewed and commented on seven to nine topics, after which one researcher (YM) made changes accordingly, acknowledging instances where multiple interpretations were possible. We provided participants with e-vouchers to compensate for their time.

## Results

### Data Statistics

After de-duplication, we obtained 365,136 tweets and 25,601 posts from the forum-based platforms (n=10,123, 39.5%, from subreddits and n=15,478, 60.5%, from Diabetes.co.uk). Among these, we identified 1765 (0.5%) tweets and 1259 (4.9%) forum posts (n=693, 55%, from subreddits and n=566, 45%, from Diabetes.co.uk) specifically posted by young people. The average number of words on Twitter and the forum-based platforms (before/after preprocessing) were 34/29 and 161/58, respectively.

### Topic Analysis

As shown in Tables 1-3, we identified 14 topics from Twitter and 10 topics from the forum-based platforms, with 7/24 (29.2%) common topics across both platforms. Financial challenges were most frequently discussed on Twitter (n=141, 18.2%), while diabetes devices were most prevalent on forum-based platforms (n=128, 24.1%). For each topic, we reported 10 representative words and 1 sentence example. Table 1 summarizes the most frequently discussed topics across platforms, providing insight into concerns, experiences, and peer interactions of the study population.

**Table 1:** Common discussion topics identified among young people with T1DM^a^ after merging on both Twitter and the forum-based platforms.

Common topics	Twitter			Forum-based platforms		
	Topic size^b^, n (%)	Selected topic words	Rephrased sentence example	Topic size^b^, n (%)	Selected topic words	Rephrased sentence example
Blood glucose management	70 (9.0)	hypo, blood, sugar, low, eat, drink, diabetic, morning, sleep, bg	“Once again, my blood sugar is somehow low after eating a lot. Ugh, there are days when I really wish type 1 diabetes didn’t exist.”	88 (16.6)	diagnose, constantly, mmol, high, hypo, normal, eat, worry, blood, struggle	“I’ve been having constant low blood sugars every night for the past week, to the point where I’ve cut my insulin dose in half, but I’m still dropping. I’m eating loads of sugar and haven’t changed my routine or diet in any way.”
Community and friendship	33 (4.3)	tweet, community, hi, support, meet, friend, family, phone, help, app	“Can we be friends? I see you're a type 1 diabetic too, so we have that in common.”	51 (9.6)	friend, talk, diabetes, type, age, understand, situation, manage, diagnose, care	“Looking for diabetic friends! Hi, I truly feel like I need friends who understand what it’s like to live with diabetes. Let me know if you’d like to connect!”
COVID-19	76 (9.8)	covid, risk, system, disease, health, diabetic, diagnose, medical, die, cure	“I’m [AGE] years old and a Type 1 diabetic, so I’m just as susceptible to Covid as someone with any other condition.”	13 (2.4)	Covid, diagnose, sick, constantly, explain, story, situation, care, worry, understand	“Hi! I’m curious about how often everyone is getting their Covid vaccine. I’m newly diagnosed with type 1 and unsure if I should keep getting vaccinated. Looking for any advice!
Diabetes and diet	81 (10.5)	carb, eat, diet, sugar, food, diabetic, bg, blood, drink, insulin	“I have type 1 diabetes, so I grew up drinking Diet Coke and never thought it was sweet enough.”	38 (7.2)	weight, diet, eat, healthy, diabete, food, gain, carb, manage, body	“I've always enjoyed a variety of foods, including the occasional treat, and I’m wondering how others manage their diet. I keep hearing that I should 'eat normally' and adjust my insulin accordingly, but I’m curious about how you structure meals—breakfast, lunch, dinner, snacks, etc.”
Diabetes devices	97 (12.5)	libre, app, phone, test, bg, system, amp, hypo, pump, insulin	“Finally, I’ve been referred to get a Libre sensor! I’m beyond excited!”	128 (24.1)	inject, dexcom, needle, mmol, injection, act, constantly, dose, unit, finger	“My Dexcom keeps reading low, but I feel fine. It says I'm low, but I don’t feel it, so I double-checked with a finger prick.! I’m not sure what’s going on with the Dexcom—it might need a change, especially since there was a lot of bleeding when I inserted it. Any advice or explanation would be appreciated!”
Emotional and psychological expression	100 (12.9)	today, morning, fuck, week, night, hate, lol, hour, friend, sleep	“Today has been good.”	124 (23.4)	constantly, hate, understand, worry, explain, care, home, fucking, sick, struggle	“Living with this disease can feel isolating, and I’ve struggled with depression since my diagnosis a year ago. I sometimes feel like no one truly understands that lonely experience. Thank you to everyone who has reached out—it honestly means so much.”
Financial challenges	141 (18.2)	diabeater (means individuals with diabetes), please, help, afford, pay, medical, supply, insulin, health, insurance	“I can’t afford my insulin and I have type 1 diabetes. Please, any help would be greatly appreciated.”	23 (4.3)	insurance, health, care, situation, advice, manage, story, job, family, act	“My insulin coverage through my parents’ insurance ended at the beginning of November, and because my college hours got cut, I had to apply for insurance on my own. I’m considering ordering from Canada through [a website]—does anyone know if it’s legitimate?”

^a^T1DM: type 1 diabetes mellitus.

^b^“Topic size” represents the number of posts within each topic and its percentage of the total number of posts from each platform.

Tables 2 and 3 summarize topics that were distinct or platform specific, providing insight into the diversity of concerns, experiences, and peer interactions in the study population.

**Table 2:** Unique discussion topics after merging identified among young people with T1DM^a^ across Twitter.

Topic	Topic size^b^, n (%)	Selected topic words	Rephrased sentence example
Advocacy and awareness	35 (4.5)	please, help, community, support, type, tweet, amp, diabeater, cure, diagnose	“Hi, I’m a type 1 diabetic and have been for a year and would be happy to participate. I’m a big advocate for how diabetes has impacted my mental health.”
Diaversaries reflection	24 (3.1)	diaversary, diagnose, diabetes, type, today, diabeater, lol, health, age, month	“Yesterday was my [YEAR] Diaversary—living with Type 1 diabetes. I’m very proud to have made it this far. Happy Diaversary to me!”
Daily life and adaption	27 (3.5)	diagnose, age, health, today, diabete, please, month, hi, help, die	“I was diagnosed with type 1 diabetes one year ago today”
Misunderstanding of T1DM	25 (3.2)	diabetic, diagnose, health, please, fuck, lol, insulin, sugar, blood, system	“That’s absolute nonsense! I’m a type 1 diabetic, and I’ve experienced both highs and lows, but I would never call someone that. It’s completely ridiculous”
Educating and raising awareness	19 (2.5)	diabete, disease, insulin, blood, sugar, please, health, cure, risk, system	“Type 1 diabetes is a completely different condition from type 2 diabetes. If you're unsure, Google the difference—it’s quite dramatic”
Insulin prescription frustration	21 (2.7)	supply, doctor, please medical, diabeater, insulin, help, health, system, pay	“I ordered some insulin on Friday, but it got denied, and I was told to call my insurance.”
Experience with health care providers	25 (3.2)	doctor, morning, diagnose, week, test, blood, medical, hospital, health, phone	“I had a phone call with my diabetes team today. They want to book an HbA1c blood test as soon as possible, and they’re happy with what I’m doing.”

^a^T1DM: type 1 diabetes mellitus.

^b^“Topic size” represents the number of posts within each topic and its percentage of the total number of posts from each platform.

**Table 3:** Unique discussion topics after merging identified among young people with T1DMa across the forum-based platforms.

Topic	Topic size^b^, n (%)	Selected topic words	Rephrased sentence example
Diabetes complications	21 (4.0)	foot, pain, diagnose, diabetic, sit, normal, type, walk, blood, hypo	“Hi, I was recently diagnosed with type 1 diabetes. My A1C is currently high, and today my legs and feet started tingling and going numb more than usual. Should I keep seeking medical attention, or is this a common symptom for people with type 1 diabetes?”
Newly diagnosed and “honeymoon” experiences	21 (4.0)	honeymoon, mmol, unit, dose, eat, level, bolus, novorapid, hypo, manage	“Hi, I’ve been diabetic for about a month now, and I think I’m in the honeymoon phase. My insulin requirements have been really low lately, even after meals with a lot of carbs. I’ve been taking just a small amount of insulin and my blood sugar levels have been normal. It’s almost like I’ve fixed my diabetes, but I’m genuinely confused. Is this normal for the honeymoon phase?”
Traveling with T1DM	24 (4.5)	advice, home, act, manage, worry, abroad, novorapid, situation, travel, hospital	“Hello! I’m hoping to go on holiday abroad next week and was diagnosed with diabetes. I’m wondering if anyone has experience traveling with diabetes and what to expect. I plan to take a cool bag for my insulin, along with supplies like testing strips, lancets, and my Libre sensor. I’m also wondering about airport procedures—should I carry a letter for my insulin and needles? Will it add extra time to security checks?”

^a^T1DM: type 1 diabetes mellitus.

^b^“Topic size” represents the number of posts within each topic and its percentage of the total number of posts from each platform.

#### Common Topics

##### Blood Glucose Management

This topic focused on the challenges of managing blood glucose levels, highlighting the unpredictability of blood sugar fluctuations and their physical and mental toll. Many described the impact on daily life and productivity, as well as the need for constant monitoring and intervention. On Twitter, the posts were more personal, immediate, and emotionally expressive (eg, ranting), capturing real-time struggles with T1DM. For example, the following post shared their “eating and overinjecting” experience during Christmas:

I had a hypo on Christmas Day, and it was awful. I ate so much and probably gave myself more insulin than I should have. Then I had to force myself to eat even more.

In contrast, the posts on the forum-based platforms were more general, focusing on broader concerns rather than in-the-moment reflection, with users consistently sharing their blood glucose values and seeking detailed information and practical strategies for blood glucose management.

##### Community and Friendship

This topic highlighted the importance of engagement and social connections within the diabetes community. On Twitter, users often introduced themselves to connect and were proud of their unique identities. Although many expressed openness to connect with others, some acknowledged challenges in maintaining these connections and sought deeper relationships. The engagement on Twitter highlighted a broader sense of community through online discussions, live streams (eg, tweet chats), and virtual events. In contrast, forum-based platform users prioritized personal, one-on-one interactions, valuing shared experiences with peers who truly understood their challenges.

##### COVID-19

This topic talked about the increased risk and anxiety experienced by individuals with T1DM during the COVID-19 pandemic. The discussions on Twitter mainly focused on the potential of their T1DM to trigger COVID-19 onset due to being immunocompromised, the unpredictability of COVID-19’s impact, the importance of vaccination and mask wearing, and advocating for public health measures. In contrast, forum-based platform users tended to focus on first-hand experiences of individuals diagnosed with COVID-19, with users sharing detailed personal narratives about their diagnostic journeys, symptom progression, and challenges in managing diabetes postinfection. Several reported developing T1DM after being diagnosed with COVID-19, despite having no prior family history or abnormal blood tests.

##### Diabetes and Diet

This topic explored dietary management strategies for T1DM. On Twitter, discussions included carbohydrate counting, beverage choices, and various dietary approaches, with young people sharing their experiences and the impact on blood glucose control. The forum-based platforms, however, primarily focused on (1) weight management with diabetes and balancing nutrition with blood glucose control and (2) sharing and seeking advice about alcohol consumption while managing diabetes, such as strategies about how to drink responsibly while balancing diabetes care, the risks of alcohol, and preferences for specific types of drinks. For example:

I’m curious about how alcohol might affect blood sugar levels if drinks are sugary.

Alcohol can lead to blood sugar dips.

##### Diabetes Devices

This topic focused on the use and management of glucose-monitoring devices, such as continuous glucose monitors (CGMs) like Libre sensors, as well as insulin pens, pumps, cannulas, and daily diabetes kits. Although many users appreciated the convenience of CGMs, they also reported challenges, such as sensor inaccuracy, device malfunctions, supply shortages, frequent maintenance, discomfort, visible marks on the skin, and difficulty finding suitable injection sites. On Twitter, users frequently shared personal experiences, enjoyment, and frustrations, as well as real-time updates about their glucose management. In contrast, forum-based platform users tended to seek more detailed advice, troubleshoot issues with glucose-monitoring devices, and also talk about finding a model that fits an active lifestyle and managing insulin needs during exercise.

##### Emotional and Psychological Expression

This section focused on the emotional and psychological impact of T1DM. On the forum-based platforms, users frequently expressed frustration, burnout, and anxiety due to the relentless demands of blood sugar management. Many described stress from unpredictable fluctuations, fears of long-term complications, and guilt over food choices. Newly diagnosed individuals often felt overwhelmed and unsupported, while others struggled with motivation and diabetes-related stigma in social and academic settings. Some found relief through structured routines, diabetes technology, and online communities, though access to mental health support remained limited. These discussions underscored the need for stronger psychological support and better education to address both the physical and emotional burdens of T1DM.

In contrast, users on Twitter focused more on daily reflections, with some wishing the day would end, while others reported more balanced or positive moments. For example:

Can today just be over? I don’t like it.

##### Financial Challenges

This topic highlighted the financial burden of managing T1DM, particularly the high cost of insulin and other necessary supplies in countries where individuals pay for their medication or rely on privately funded health care. Individuals discussed difficulties in affording treatment and seeking financial assistance. Many sought advice on finding affordable insurance or alternative solutions when their coverage was inadequate.

#### Unique Twitter Topics

##### Advocacy and Awareness

This topic focused on advocacy and awareness efforts for T1DM. There was a strong emphasis on using social media and events to share knowledge, participate in fundraising, and dispel misconceptions about this condition—for example, hashtag campaigns such as *#InsulinForAll* and *#WorldDiabetesDay.*

##### Diaversaries Reflection

This topic revolved around personal reflections on *diaversaries*, the anniversaries of a T1DM diagnosis. Key discussions included the challenges in and resilience required to manage the condition. Some messages celebrated personal growth, perseverance, and a sense of acceptance and pride in how they have adapted (eg, *Happy 10 year #diaversary to me. #10YearDiaversary*), while others highlighted the struggles and life changes diabetes has brought.

##### Daily Life and Adaptation

This topic covered the process of diagnosing T1DM and the ongoing adaptation to life with the condition. It included reflections on diagnosis anniversaries and the long-term impact on personal development. For example:

A year ago today, I was diagnosed with Type 1 diabetes. What a life it’s been! I’m very proud of where I am and how I’ve handled things. I won’t let my disease control how I live.

##### Misunderstanding of T1DM

This topic reflected the frustrations of young people with T1DM, particularly regarding unsolicited advice, misunderstandings, and stereotypes. Many expressed the emotional strain of constantly addressing misconceptions, responding to virtue signaling, and repeatedly explaining their condition to others who had limited knowledge of diabetes. For example:

It can be frustrating when I try to explain what I can or can't eat, just because they know someone with the condition.

##### Educating and Raising Awareness

This topic focused on educating others about T1DM, particularly by distinguishing it from type 2 diabetes. The main themes included raising awareness, correcting misconceptions, and encouraging curiosity and learning. Unlike the topic “misunderstanding of T1DM” that shared personal struggles, this topic was specifically centered on providing clarification and promoting accurate information about T1DM. For example:

Hi! If you have any curiosity or questions about type 1 diabetes, feel free to ask me!

##### Insulin Prescription Frustration

This topic highlighted the administrative and systemic challenges faced by young people with T1DM in accessing essential insulin and related care. They reflected frustrations with mismanaged prescriptions, inadequate health care support, bureaucratic hurdles, and the dire consequences of such oversights. For example:

Is it a problem if my GP refuses to put insulin on my prescription and instead insists I use their online form to request it? Obviously, it just makes my life harder by forcing me to order what’s on my repeat via the NHS app and find another way to get my insulin.

##### Experience With Health Care Providers

These posts discussed individuals’ experiences with diabetes care, from positive and reassuring interactions with health care providers to frustrations with administrative issues and poor communication. Many highlighted the need for understanding and compassionate care, noting that “half the battle was won” when their providers truly understood them. Others reported negative experiences, such as being treated like a child. Posts also highlighted difficulties navigating the health care system, such as finding appointment letters.

#### Unique Forum-Based Platform Topics

##### Diabetes Complications

This topic focused on diabetes-related complications, particularly neuropathy and gastrointestinal issues. Young people shared their experiences with foot pain, burning sensations, and strategies for managing neuropathy and foot discomfort. They also discussed managing stomach issues, such as nausea, pain, and appetite loss related to diabetes and its complications.

##### Newly Diagnosed and “Honeymoon” Experiences

This topic focused on young people who were newly diagnosed with diabetes and their experience during the “honeymoon period”. During this phase, they observed a temporary decline in insulin needs, sometimes resulting in normal or low blood sugar levels, even after consuming carbohydrates. Many expressed confusions about the duration and nature of this period, therefore frequently seeking advice on adjusting to their new reality, understanding treatment protocols, and managing the early stages of diabetes care.

##### Traveling With T1DM

In this topic, individuals focused on seeking advice for managing diabetes while traveling, including tips for carrying supplies, navigating security checks, and maintaining routines abroad.

## Discussion

### Principal Findings

This study used a novel topic modeling approach to investigate young people’s actual use of different social media platforms for T1DM self-management. The analysis identified 24 topics: 14 topics from Twitter and 10 from forum-based platforms, with 7 common topics across both.

Our findings indicate both similarities and differences in how young people engage with Twitter and the forum-based platforms for T1DM self-management. Users on all platforms discuss topics such as blood glucose management, friendships and community, diet, diabetes monitoring devices, emotional and psychological expression, COVID-19, and financial challenges. However, for the same topic, Twitter posts are more immediate and experience driven, with users sharing personal milestones, quick troubleshooting tips, and brief reflections on daily challenges. In contrast, the forum-based platforms foster more in-depth interactions, where users seek and provide comprehensive advice, particularly for problem solving.

Beyond these general discussions, users tend to use Twitter for advocacy, public education, raising awareness, diagnosis anniversary reflection, sharing experiences with insulin prescriptions, and engaging with health care providers. Meanwhile, the forum-based platforms serve as a space for practical guidance, particularly for newly diagnosed individuals who seek advice on managing complications and using diabetes devices while traveling.

### Relation to Other Studies

#### Confirmative Findings Compared to Previous Qualitative Research

Several of our findings align with previous qualitative research on the challenges young people face in managing T1DM. For example, our findings on blood glucose management echo studies [52,53] that emphasize the constant need for self-monitoring, the struggles with hypoglycemia and hyperglycemia, and the emotional weight of maintaining stable blood sugar levels. Young people in our social media analysis shared similar concerns about the impact of blood sugar fluctuations on their social life, school, and physical activities, reinforcing existing evidence on the pervasive influence of diabetes management on daily life.

Furthermore, our findings align with social support theory [54,55], highlighting how shared learning and peer engagement can support self-management practices. For example, our findings support prior research that identified social media as crucial avenues for informational and technical support essential for chronic disease management [56,57]. We identified that young people frequently seek and share practical tips and technical support on managing blood glucose, troubleshooting diabetes devices, and maintaining healthy blood sugar levels through diet, reinforcing the importance of social media in their diabetes management. In addition, our findings about emotional expression, and community and friendship are consistent with the literature identifying online communities as essential sources of emotional and peer support for young people with T1DM [21,28,31]. Young people share their feelings on social media and seek comfort and emotional support. Social media platforms enable peer interactions that reduce feelings of isolation and foster a sense of belonging among those facing similar challenges.

Moreover, our findings indicate that young people’s interactions with traditional health care systems, such as insulin prescriptions, diabetes reviews, and appointments, are not always positive experiences. This mirrors previous research suggesting a need for greater integration of traditional health care support within social media platforms to offer more accessible and tailored assistance to this particular population [31].

Our study also highlighted the importance of public awareness and educating others about T1DM. Young people expressed frustration with common misunderstandings; misconceptions, such as confusing type 1 diabetes with type 2 diabetes; and stereotypes linking T1DM, such as being overweight. These findings echo previous research on the barriers young people with T1DM face in managing societal misunderstandings about their condition [28].

#### New Findings Compared to Previous Qualitative Research

Apart from these common discussions, our study uncovered several topics that have been less discussed in previous qualitative research on young people’s experiences with T1DM. This demonstrates the value of analyzing social media data through topic modeling to capture emerging concerns and social media–specific practices.

Although previous studies have explored the feasibility of using social media or telemedicine for delivering T1DM health care [58-61] during the COVID-19 pandemic, the specific impact of the pandemic on young people with T1DM has received less attention. In this study, we identified COVID-19 as a prominent topic of discussion, highlighting increased vulnerability and anxiety related to the virus. Although clinical studies have examined the relationship between T1DM and COVID-19 [62-64], our analysis of real-time social media conversations captured the lived experiences and emotional responses in authentic detail that traditional qualitative methods relying on memory and self-reporting might miss. We identified that the pandemic did not just create new concerns but also transformed existing ones, intensifying anxieties about health vulnerability and disrupting established care routines.

Additionally, social media–driven advocacy emerged as another prominent topic, particularly around the hashtag campaigns like *#InsulinForAll* that call attention to the challenges of accessing insulin and the need for greater public awareness. These online campaigns represent collective action that transcends individual experiences typically captured in interview-based studies. Our findings reveal how young people with T1DM leverage social media not, just for support, but also as a platform for systemic change that is rarely documented in traditional qualitative research.

Furthermore, the reflection of diaversaries represents a community-specific cultural practice that has received minimal attention in previous qualitative research. Young people with T1DM reflected on their diaversaries day for celebrating resilience and reflecting on the challenges they have faced, revealing a cultural phenomenon unique to online T1DM communities. By capturing these practices, our topic modeling approach revealed how young people reframe their diagnosis narratives from purely medical experiences to important life milestones worthy of reflection.

### Limitations

One limitation of our study is that the data extraction was conducted over different time frames due to the API limitations on Twitter and Reddit. This may have introduced potential bias, as current events may have affected the topics of discussion during these periods, particularly during the COVID-19 pandemic.

In addition, our analysis was limited to three platforms (Twitter, Reddit, and Diabetes.co.uk). As stated in the *Methods* section, we did not include platforms more popular among young people, such as TikTok, Snapchat, and Instagram [65]. We acknowledge that this may have limited our ability to fully capture the diverse perspectives of young people with T1DM, particularly those in younger demographics who are more likely to use these alternative platforms. Furthermore, data from publicly posted content may overrepresent those who are relatively active and comfortable in openly discussing their condition, while overlooking the perspectives of users who use social media for self-management but without posting. This means our findings might not represent the full breadth of actual use patterns of young people with T1DM. Furthermore, our design did not allow us to capture views from those who do not use social media, for example, because they are not interested, are too unwell, or do not have access. Future research could consider incorporating a wider range of social media platforms, developing methods to capture the perspectives of less publicly vocal participants, and using complementary approaches to include nonusers.

Another limitation arose from the topic model method we used. Although CAST could effectively identify large-scale topical patterns, it represents each document by its most dominant topic. This approach may have inadvertently filtered out less frequently discussed but potentially important topics, leading to an incomplete picture of the full range of discussions. Incorporating more granular-level analysis (eg, truncating large documents into paragraphs or sentences) in the future could lead to more nuanced topic identification and provide a more comprehensive understanding of social media content related to T1DM management.

Despite analyzing discussion topics related to T1DM self-management, young people’s attitudes toward the same topics may vary and remain poorly understood. For example, some celebrated personal growth on diaversaries, while others highlighted the struggles and life changes associated with T1DM. Furthermore, online exchanges are not always supportive and can sometimes be negative or harmful. Conducting a sentiment analysis could help clarify these variations and provide a better understanding of how safe and supportive social media spaces are for young people with T1DM.

### Implications for Practice and Research

The study demonstrated that topic modeling of social media data can complement traditional qualitative approaches by capturing emergent concerns, collective action, and community-specific practices that might be overlooked in interview-based studies. For researchers, this supports using topic modeling in mixed methods studies to better track patient needs over time. Future research could build on this work by directly comparing how young people describe their social media use in interviews, with patterns observed in actual usage data through topic modeling. Such comparisons may deepen our understanding of the gaps between self-reported and actual behavior patterns.

The study highlighted that young people with T1DM engage with Twitter and forum-based platforms in different ways, with Twitter discussions being more immediate and experience driven, focusing on daily reflection, advocacy, awareness campaigns, and financial support, while forum-based platforms emphasize more in-depth discussions, where users seek and provide comprehensive advice, troubleshooting, and sustained peer support. These differences could inform targeted social media strategies. Practitioners could consider using Twitter for real-time outreach and engagement, while using forums to foster peer learning communities and deliver structured self-management support. Aligning interventions with platform-specific strengths may improve the reach and impact of digital support for T1DM self-management.

The pipeline applied in our paper could provide a transparent and reproducible framework for topic extraction and summarization in the future. Although recent approaches have explored directly prompting LLMs to identify topics [47,48], these models often operate in an agnostic manner and lack the interpretability required for robust topic analysis. Moreover, LLMs are prone to hallucinations [66,67], generating plausible yet inaccurate information, which poses additional challenges for reliable topic modeling. Instead, we used CAST, a Sentence-BERT-based model that enables interpretable clustering of semantically similar sentences and facilitates the selection of representative sentences for each topic. LLMs were instead used for summarization tasks via a simple prompting strategy, where their strengths in contextual synthesis and natural language generation are most effective. A human-in-the-loop process (involving YM and PPIs) was incorporated to ensure that the generated summaries accurately reflect the perspectives of young people.

### Conclusion

Our paper used topic modeling to identify topics by young people with T1DM discussed on social media platforms, with LLMs and patient input aiding the interpretation of topics. Our findings reinforce the challenges and emotional burdens faced by young people with T1DM, social media’s role in providing informational and emotional peer support, and the need for educating others and providing more accessible and tailored health care. Topics such as COVID-19 impacts, advocacy initiatives, and diaversaries reflection are more frequently discussed in social media discussions than they have been in prior qualitative studies. Platform-specific patterns are also evident, suggesting that practitioners could consider the differences when designing digital interventions to ensure the delivery method aligns with the communication style and support needs of the target audience.

## Data Availability

All the code in this paper, including data extraction, data preprocessing, and topic identification, are available in GitHub [68].

## Appendix

Multimedia Appendix 1 Regular expression patterns.

Multimedia Appendix 2 Topic quality evaluation.

## Abbreviations

API: application programming interface
CGM: continuous glucose monitor
LLM: large language model
PPI: patient and public involvement
T1DM: type 1 diabetes mellitus
