# Supplementary material for: Investigating Social Media Use by Young People to Self-Manage Type 1 Diabetes Mellitus: Large-Scale Analysis of Social Media Discussions Using Topic Modeling
Source: J Med Internet Res. 2025 Oct 20;27:e78632. doi: 10.2196/78632 (PMC12583938; doi:10.2196/78632)
Supplement: Multimedia Appendix 1 [file jmir_v27i1e78632_app1.doc]

## **Multimedia Appendix 1.** **Regular expression patterns**

For Twitter and Reddit, we used rule-based methods to detect their self-reported age information from their tweets/posts which matched the regular expression patterns (see Table 3).

Table 3. Rule-based age patterns and examples.

| **Patterns** | **Examples** |
| --- | --- |
|  |  |
| *Turning or turned <two digits>* | *“I’m turning 25 next month.”* |
| *born (in|on) <four- or two-digit year | day, month, year>* | *“I was born in 2008 and diagnosed with type 1 diabetes at age 7.”* |
| *I am <number> (years|yrs|yo)* | *“I am 25 years old and have been living with type 1 diabetes for 15 years.”* |
| *(I’m|I am) <number>* | *“I’m 20 and recently started using a continuous glucose monitor for my type 1 diabetes.”* |
| *(I’m|I am) now <number>* | *“I am now 14 and excited to join a type 1 diabetes camp this summer.”* |
| *As a <number> year* | *“As a 18 year old student, I hate …”* |
| *<number> in <four-digit year>* | *“I was 16 in 2015 when I switched from injections to an insulin pump.”* |
| *<number> (years | yrs) old or young* | *“I am a 24 years old young man who’s been living with type 1 diabetes for over 10 years.”* |
| *(M|F) number> or <number>(M|F)* | *“25F with T1 looking for friends online.”* |
| *(I|I’m|me|my) [<number>] (M|F)* | *“I’m 24M and have had type 1 diabetes since I was a teenager.”* |
| *<number>-year-old* | *“As a 15-year-old girl, I use pump every day to manage my blood sugar.”* |
