# Supplementary material for: Investigating Social Media Use by Young People to Self-Manage Type 1 Diabetes Mellitus: Large-Scale Analysis of Social Media Discussions Using Topic Modeling
Source: J Med Internet Res. 2025 Oct 20;27:e78632. doi: 10.2196/78632 (PMC12583938; doi:10.2196/78632)
Supplement: Multimedia Appendix 2 [file jmir_v27i1e78632_app2.docx]

## **Multimedia Appendix 2.** **Topic quality evaluation**

Topic coherence (TC) and topic diversity (TD) are critical metrics for evaluating topic models. A robust topic model should generate topics that are both coherent and diverse. In our experiment, we used normalised point-wise mutual information (NPMI) [1] to assess TC and the percentage of unique words among topic words to evaluate TD [2]. Additionally, we adapted large language model-based metrics, LLM-TC and LLM-TD, to mimic the human evaluation process. We employed a 4-point rating scale prompt as defined in CAST (Section 3.3) [3], which has demonstrated a high Pearson correlation with human judgment.

NPMI measures word co-occurrence within the corpus and ranges from -1 to 1. TD ranges from 0 to 1. LLM-TC and LLM-TD range from 0 to 4. To facilitate comparison, we normalized all data to [0,1] using min-max normalization, with higher scores representing better performance.

For a given value from the original range , the normalized value in the target range is calculated as:

CAST has shown improved performance over baseline models in generating more coherence and diverse topics on BBC News, 20NewsGroups and Elon Musk’s tweets as shown in CAST [3]. To validate the use of CAST on our dataset, we conducted further examination with LDA. Results (see Table 4) show that CAST outperformed LDA in most cases across the datasets except the TC on Twitter.

Moreover, CAST can provide sentences associated with each topic, which, alongside topic words, offer a more comprehensive understanding of the topic's meaning. In contrast, LDA relies solely on topic words, which may require prior knowledge of the corpus for accurate interpretation. Therefore, we employed CAST as our topic modelling approach in this paper.

Table 4. Normalized model evaluation results with best values underlined. TC represents Topic Coherence. TD represents Topic Diversity. LLM- represents Large Language Model-based metrics.

| **Models** | **Twitter** | | | | **Forum-based platforms** | | | |
| --- | --- | --- | --- | --- | --- | --- | --- | --- |
|  | TC | LLM-TC | TD | LLM-TD | TC | LLM-TC | TD | LLM-TD |
| LDA | 0.49 | 0.8 | 0.22 | 0.5 | 0.5 | 0.68 | 0.23 | 0.25 |
| CAST | 0.47 | 0.93 | 0.32 | 0.75 | 0.5 | 0.71 | 0.39 | 0.75 |

## References

1. Bouma G. Normalized (Pointwise) Mutual Information in Collocation Extraction. Proc Bienn GSCL Conf 2009 2009 Jan 1;

2. Dieng AB, Ruiz FJR, Blei DM. Topic Modeling in Embedding Spaces. Trans Assoc Comput Linguist 2020 Jul 1;8:439–453. doi: 10.1162/tacl_a_00325

3. Ma Y, Xiao C, Yuan C, Veer SNVD, Hassan L, Lin C, Nenadic G. CAST: Corpus-Aware Self-similarity Enhanced Topic modelling. In: Chiruzzo L, Ritter A, Wang L, editors. Proc 2025 Conf Nations Am Chapter Assoc Comput Linguist Hum Lang Technol Vol 1 Long Pap Albuquerque, New Mexico: Association for Computational Linguistics; 2025. p. 7548–7561. doi: 10.18653/v1/2025.naacl-long.386
